# Clinical Robotic Surgery Association Fifth Worldwide Congress, Washington DC, 3–5 October 2013: Robotic Colorectal Surgery

**DOI:** 10.3332/ecancer.2014.385

**Published:** 2014-01-13

**Authors:** Paolo Pietro Bianchi, Alessio Pigazzi, Gyu Seog Choi

**Affiliations:** 1 European Institute of Oncology, Milan 20141, Italy; 2 University of California, Irvine, CA 92697, USA; 3 Kyungpook National University, Daegu 702–701, Korea

**Keywords:** robotic surgery, colorectal cancer, transrectal surgery, Clinical Robotic Surgery Association

## Abstract

The colorectal session was one of the most successful and well attended sessions at the Fifth Worldwide Clinical Robotic Surgery Association Congress because of the increasing interest and diffusion of robotic techniques in this specific field. This session was structured as follows: two technical focuses, one on rectal resection and the other on right colectomies; a journal club with two hot topic papers presented by the authors; a face-to-face on single-port laparoscopic versus robotic surgery; an update on the transanal approach; and three lectures, on the oncologic safety of robotic total mesorectal excision, on the use of fluorescence in colorectal surgery, and finally an update on the ongoing ROLARR trial (laparoscopic versus robotic rectal resection).

## Technical focus on right colectomies

In the focus on right colectomies, Giuseppe Spinoglio (Alessandria, Italy) and Gyu-Seong Choi (Daegu, Korea) compared their robotic techniques. The main issue for both surgeons was the necessity to perform a complete mesocolic excision with a high ligation of the vessels, in order to obtain the highest number of lymph nodes retrieved as possible and a safe oncologic resection. Spinoglio performs a full robotic technique with a medial-to-lateral approach and a complete exposure of the left border of the superior mesenteric vein, so to dissect the vessels at their origin. The ileocolic anastomosis is performed intracorporeally with a hybrid stapling and sewing technique. In addition, Spinoglio underlined the advantages of the robot during the vascular dissection and during the step of the anastomosis, because of the improved dexterity of the endowrist robotic instruments during the intracorporeal sutures. Dr Choi’s approach is different, starting with the mobilisation of the root of the mesentery until the duodenum and exposing the vessels from a lateral to medial direction, the anastomosis is performed intracorporeally with a full stapling technique. Based on the result of a recent randomised clinical trial in their institution, Dr Choi’s opinion is that the robotic system does not add particular advantages in right colon resections and, because of the high costs of the instrumentation, he does not suggest the routine use of the robot in this specific operation.

## Journal club

In the journal club session, two papers published in 2013 were discussed: the first was a randomised control trial on robotic versus laparoscopic right colectomy, published in the *British Journal of Surgery *by Prof Jun Seok Park (Daegu, Korea). Henry Lujan (Miami, United States) and Luca Giordano (Philadelphia, United States) were the participants in the discussion. The study is a randomised two-arm control trial comparing the two different techniques with 71 patients in total. The authors conclude that robotic right colectomy does not provide significant advantages compared with the laparoscopic technique in term of short-term outcomes and oncologic safety, but the costs are higher than laparoscopy and its use is not routinely recommended. The discussion participants underlined the possible bias correlated to the different number of intra and extracorporeal anastomoses in the two arms, with a higher number of intracorporeal in the robotic group, and they suggested the necessity of long-term outcomes results to be more conclusive.

The second paper was presented by Fabrizio Luca (Milan, Italy): ‘Impact of Robotic Surgery on Sexual and Urinary Functions After Fully Robotic Nerve-Sparing Total Mesorectal Excision for Rectal Cancer’ (*Annals of Surgery, *2013). The participants in the discussion were Dr Choi and Dr Marecik (Chicago, United States). The study is a prospective single-centre series of 74 patients and evaluates the impact of robotic surgery for rectal cancer on sexual and urinary functions in male and female patients. Dr Luca concluded that robotic nerve-sparing total mesorectal excision (TME) allows for better preservation of urinary and sexual functions when compared with data from the literature on laparoscopic and open procedures. The discussion participants underlined the absence of a preoperative evaluation of the urinary and sexual functions, and the necessity of a randomised control trial. All the experts agree on the lack of conclusive data in the literature compounded by the differences in the questionnaires and the difficulties in collecting data.

## Face-to-face

The face-to-face was focused on single-site procedures, comparing laparoscopy with curved instruments and robotic surgery. Dr Dapri (Brussels, Belgium) presented a right colectomy performed with special curved laparoscopic instruments designed by him and dedicated to this operation. The approach was through a suprapubic incision, with the insertion of a single-site device and two 5-mm trocars, positioned laterally to the device, but through the same 2.5-cm incision. The anastomosis was performed intracorporeally with a hybrid stapling and hand sewn technique. The oncologic results in terms of specimen length and the number of lymph nodes retrieved were adequate. The clinical outcome was uneventful with an excellent cosmetic result. Dr Obias (Washington DC, United States) uses a single-port device not specifically dedicated to the DaVinci system and connects two robotic arms to the robotic trocars inserted in the device. He performed different procedures with this system as cholecystectomy, right and left colectomies. The main advantage of the robot is the opportunity to invert right and left ‘hands’ and avoid the cross of the instruments, sometimes necessary in the laparoscopic single-port technique. Both the speakers underlined the feasibility of the techniques presented in selected cases and that the major advantage of the single-port surgery is the reduction of scares and parietal complications correlated to the multiport procedures. Furthermore, in the case of intraoperative problems, it is possible to convert in a standard laparoscopic procedure, maintaining all the advantages of minimally invasive surgery.

## Update on transanal approach

In this session, the innovative transanal and transrectal techniques, which have quickly developed in recent years, were analysed. Dr Patricia Sylla (Boston, United States) is one of the pioneers of the TME performed through a transrectal approach. The aim of this approach is to dissect the lower part of the rectum from below with an endoluminal incision of the rectal wall. The transanal approach is performed with standard laparoscopic instruments inserted through a single-port device and with the insufflation of the rectum with carbon dioxide, in order to maintain an adequate field view. The advantage of the procedure is mainly anatomical, because of the easy access through the rectal wall at the perirectal planes, which usually is one of the most challenging steps of the rectal resection. Through this, surgical plane is possible in some cases to perform also the other steps of the rectal resection as inferior mesenteric vessels ligation and taken down of the left colonic flexure, in other more complex cases, these two steps are performed laparoscopically. The specimen is removed transanally and the coloanal anastomosis is performed with a circular stapler. The initial experience, shared also with the group of Antonio Lacy (Barcelona, Spain), demonstrates an oncologic safety of the procedure and good short-term clinical outcomes, avoiding any large abdominal wall incision, rather than the 12-cm maximum scars of the trocars insertion. Dr Choi showed his experience in transanal removal of the specimen during robotic rectal resection. The technique is a hybrid laparoscopic and robotic procedure, the vascular dissection and the splenic flexure mobilisation are performed with standard laparoscopic instruments, instead the TME is performed with the robot assistance. When TME is completed the rectum is transected with robotic shears and the specimen is removed transanally. The anastomosis is performed with a circular stapler after the closure of the rectum around the stapler with a purse string suture done robotically and reinforced with a laparoscopic endoloop. Prof Choi underlined the necessity to follow an accurate patient selection, avoiding advanced stages with bulky tumours. The advantage of this procedure is the absence of abdominal wall incisions to remove the specimen and sometimes the possibility to perform a sewn anastomosis, using the dexterity of the robotic instruments. Finally, Sergio Larach (Orlando, United States) presented the wide experience of Florida Hospital in transanal minimally invasive surgery (TAMIS) for local excision and more extended procedures. Dr Larach demonstrates how TAMIS techniques can be used to perform retrograde protectomy with the DaVinci system, showing the modalities to use the robot arms in transrectal operations. The conclusion of Dr Larach was that TAMIS is an advanced platform that provides a safe and effective method to remove benign neoplasms, as well when carefully selected, early stage malignancies of the mid and distal rectum.

## Technical focus on rectal resection

In this session, Dr Marecik illustrated the technical details of full robotic rectal resection. The robotic system can be used in all the steps of the procedure, and if necessary, the docking of the robot can be changed moving from the upper to the lower quadrants of the abdomen without an overall waste of time. The high definition three-dimensional vision and the stability of the camera of the DaVinci robot are helpful in all the steps of the operation, especially in the narrow space of the pelvis. Dr Marecik presented very high-quality videos showing some cases of robotic rectal resections also in advanced rectal cancer and stressed how these advanced stages can be approached and concluded with a low risk of conversion to open surgery because of the robotic system.

## Three lectures concluded this session

### Oncologic safety of robotic rectal resection

Dr Bianchi (Milan, Italy) presented an update of the current data on robotic rectal surgery. At the moment, the studies comparing laparoscopic versus robotic rectal surgery are mainly single-centre series not randomised and with a low level of evidence. Despite the low volume of data, several meta-analyses have been published, and all of the studies demonstrated a lower rate of conversions to open surgery in the robotic group when compared with standard laparoscopy. Furthermore, the pathologic results in terms of clear margins of resection and number of lymph nodes harvested demonstrated an oncologic safety of the robotic procedure at least in the short-term period. The low rate of conversion to open surgery of robotic rectal resection is attributed by all the authors to an attenuated learning curve of the technique and the technical advantages of the robotic instruments in the pelvic space. In summary, Dr Bianchi said that robotic surgery is a promising technique that could increase the diffusion of minimally invasive rectal surgery, which is still too low worldwide, and has a great potentiality in terms of teaching because of the dual-console system. The main concerns related to the robotic method are the high costs of the acquisition and maintenance of the equipment with respect to a lack of evidence of better results than standard laparoscopy.

### Utility of indocyanine green fluorescence imaging in colorectal surgery

Dr Hellan (Ohio, United States) presented the use of indocyanine green (ICG) fluorescence imaging with the DaVinci system. This ICG dye is injected intravenously during the operation and the use of a near-infrared light allows a real-time visualisation of the vascular perfusion of the colonic stumps. The images are very clear, and in case of a bad vascularisation, the colonic stump does not pick up green fluorescence. Dr Hellan stressed the necessity to evaluate the vitality of the anastomotic tissue in order to prevent postoperative leakages, and the ICG fluorescence imaging is an easy and safe method to evaluate the anastomosis intraoperatively. A further application of ICG imaging in colorectal surgery under evaluation is for lymphatic mapping, to identify sentinel lymph nodes and possibly improve staging of colorectal disease

### Update on ROLARR trial

Alessio Pigazzi (Irvine, United States) presented an update on the ongoing trial of robotic versus laparoscopic rectal surgery (ROLARR). This trial is a randomised two-arm control trial; the primary endpoint is the rate of conversions to open surgery, the key secondary endpoints are circumferential resection margin positivity and local recurrence, and other secondary endpoints are safety of the technique, functional results, quality of life, and economics considerations. The ROLARR trial is a worldwide study involving 28 centres in the United States, Europe, Asia, and Australia. The total recruitment as of 30 September 2013 is 257 patients, and the expected end of recruitment is at the end of 2014 , with the final enrollment expected to reach 400 cases.
